# Annexin A2 interferes with complement regulation within the glomerulus

**DOI:** 10.1016/j.jbc.2025.110657

**Published:** 2025-08-30

**Authors:** Brandon Renner, Jennifer Laskowski, Felix Poppelaars, Russell Whelan, Kyrie Milliron, Vojtech Petr, Judith Blaine, Yunus Ozekin, Viviana P. Ferreira, Matthew C. Pickering, Christoph Q. Schmidt, Katherine Hajjar, Anne Davidson, Joshua M. Thurman

**Affiliations:** 1 Department of Medicine, University of Colorado School of Medicine, Anschutz Medical Campus, Aurora, Colorado, USA; 2 Department of Pediatrics, University of Colorado School of Medicine, Anschutz Medical Campus, Aurora, Colorado, USA; 3 Department of Nephrology, Institute for Clinical and Experimental Medicine, Prague, Czech Republic; 4 Department of Medical Microbiology and Immunology, University of Toledo College of Medicine and Life Sciences, Toledo, Ohio, USA; 5 Centre for Inflammatory Disease, Department of Immunology and Inflammation, Imperial College, London, United Kingdom; 6 Institute of Experimental and Clinical Pharmacology, Toxicology and Pharmacology of Natural Products, University of Ulm Medical Centre, Ulm, Germany; 7 Department of Pediatrics, Weill Cornell Medicine, New York, New York, USA; 8 Institute of Molecular Medicine, Feinstein Institutes for Medical Research, New York, New York, USA

**Keywords:** Annexin A2, complement, factor H, kidney, inflammation

## Abstract

The alternative pathway of complement is an important pathogenic driver of a variety of glomerular diseases. Factor H is a soluble complement regulatory protein, and it is known to play a critical role in protecting the kidney from alternative pathway-mediated injury. Other proteins, however, can interfere with complement regulation by Factor H, thereby predisposing the kidney to injury. Annexin A2 was previously shown to bind to Factor H and is expressed by several resident cell types in the kidney. In the current study, we show that extracellular annexin A2 binds to the region of Factor H encompassing short consensus repeats 6 to 8, impairing the ability of Factor H to regulate complement activation on the surface of glomerular endothelial cells and podocytes *in vitro* and *in vivo*. Annexin A2 does not, however, impair Factor H function on extracellular matrix or guinea pig erythrocytes. Targeted deletion of annexin A2 in mice attenuates cyclosporine-induced kidney injury in mice and deficiency of annexin A2 expression reduces complement activation on the surface of extracellular vesicles released from endothelial cells in this model. Review of publicly available kidney transcription datasets revealed that annexin A2 is expressed by several cell types in the kidney, and that expression is increased in multiple different disease states. Annexin A2, therefore, may serve as an intrinsic “positive regulator” of complement activation in the kidney, promoting the inflammatory response after various kidney insults.

A large body of preclinical and clinical research has identified the alternative pathway of complement as a key driver of several kidney diseases, including atypical hemolytic uremic syndrome (aHUS), C3 glomerulopathy (C3G), anti-neutrophil cytoplasmic antibody (ANCA) associated vasculitis, IgA nephropathy, and focal segmental glomerulosclerosis (1, 2, 3, 4, 5). Congenital and acquired defects in the body’s ability to regulate the complement system are strong risk factors for these diseases, and increased complement activation is associated with a worse prognosis in prevalent patients (2, 6, 7, 8). Impairments in the ability to control the alternative pathway can be caused by loss-of-function variants in genes for the complement regulatory proteins, gain-of-function variants in complement proteins involved in activation (C3 and factor B), autoantibodies that impair the function of the regulators, or autoantibodies that stabilize the convertases (9, 10).

The unique susceptibility of the kidney to complement-mediated injury may be due, in part, to the critical role played by Factor H in regulating the alternative pathway in glomerular capillaries. Although multiple regulatory proteins are expressed on all cells throughout the body (11), an isolated deficiency of Factor H is sufficient to cause alternative pathway activation on glomeruli in animal models and in humans (12, 13). Most patients affected by complement-mediated kidney diseases do not have specific molecular defects in Factor H function or expression; however, this indicates that Factor H, even when it is fully functional, can be overwhelmed during disease. One mechanism by which regulation by Factor H can be subverted involves expression of proteins that antagonize it and act as “positive regulators” of the complement system (14). When present in the kidney, these proteins lower the threshold for alternative pathway activation in the glomerulus (15). The five Factor H-related proteins (FHRs), for example, are structurally related to Factor H but lack its complement regulatory domain (16, 17). These proteins may competitively block Factor H from binding to tissues (18, 19, 20), and higher circulating levels of the FHRs are associated with a worse prognosis in several glomerular diseases (6, 7, 19, 21).

Another mechanism by which proteins can modulate Factor H is through protein−protein interactions that involve its binding regions [short consensus repeats (SCRs) 6–8 or 19–20]. Each SCR is approximately 60 amino acids in length and contains two internal disulfide bonds. We previously reported that annexin A2 (AnxA2) directly binds to Factor H and limits its ability to regulate alternative pathway activity on tubular epithelial cells (22). The annexins are a family of 12 structurally related proteins (numbered 1–11 and 13 in humans) with a diverse range of functions (23, 24). Each of the annexins has a distinct N-terminal domain, but all members of the annexin family contain four homologous alpha helix domains that bind to phospholipids and membranes in a Ca^2+^-dependent manner (24, 25, 26). AnxA2 has multiple reported intracellular and extracellular functions, including protein-protein interactions with several ligands (25, 27, 28, 29, 30, 31, 32, 33). Notably, AnxA2 is expressed by multiple cell types within the kidney, including glomerular endothelial cells (GEnCs), mesangial cells, podocytes, and tubular epithelial cells (34, 35, 36, 37).

Given the susceptibility of the kidney to impairments in Factor H function as well as the broad expression of AnxA2 by resident kidney cell types, we hypothesized that local upregulation of AnxA2 may be an important mechanism by which kidney insults engage the alternative pathway of complement and promote inflammation. To further explore the role of AnxA2 in healthy and diseased kidneys, we examined the interaction of AnxA2 with Factor H and the effects of AnxA2 on complement regulation/activation on the surface of specific glomerular cell types. We also tested whether overexpression or underexpression of AnxA2 affects complement activation in the kidney. Finally, using preclinical and clinical datasets, we examined the expression of AnxA2 in several kidney diseases.

## Results

### The third and fourth core domains of annexin A2 bind to SCRs 6 to 8 of factor H

**Figure 1:**
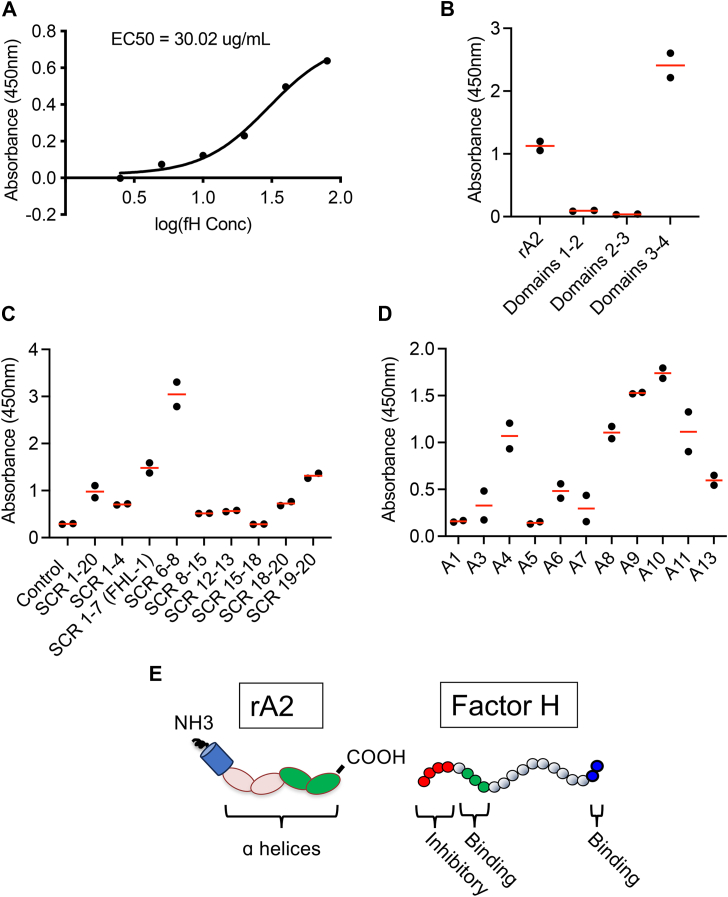
**Direct binding of Factor H and annexin A2.***A*, varying concentrations of Factor H was immobilized on an enzyme-linked immunosorbent assay (ELISA) plates, and a fixed concentration of recombinant annexin A2 (rA2) was added to the wells. Bound annexin A2 was detected with an antibody, demonstrating dose dependent binding of the proteins. *B*, the same reaction was performed with recombinant fragments encompassing core domains 1–2, 2–3, or 3–4. Factor H bound to full sized rA2 and the 3–4 core domain peptide. *C*, we examined the binding of rA2 to peptide fragments of Factor H incorporating various short consensus repeats (SCRs). The strongest binding was seen to SCRs 6–8. *D*, the binding of Factor H to annexins 1–13 was examined in the ELISA assay. The signal was above background for all annexins, and it was strongest for annexin A10. *E*, depictions of structural domains in the annexin A2 and Factor H proteins. The binding regions of each protein are shaded in *green*.

We generated recombinant AnxA2 (rA2) and recombinant murine Factor H, and we confirmed that the two proteins bound to each other in enzyme-linked immunosorbent assays (ELISAs; Fig. 1*A*). We also generated fragments of murine AnxA2 and examined which regions of the protein were necessary for binding to Factor H (Fig. 1*B*). A peptide fragment incorporating the third and fourth core domains of AnxA2 bound to Factor H, whereas the other regions of AnxA2 did not bind. We next tested the binding of rA2 to fragments of Factor H. The strongest binding was seen with the SCR 6 to 8 region (Fig. 1*C*), consistent with a previous report (38). Accordingly, the alternative splicing variant of Factor H, Factor H-like protein 1 (FHL-1; containing SCRs 1–7), also bound to rA2. SCR19 to 20 are also bound, but to a lesser degree. Given the structural similarities core domains among the annexins, we tested the ability of Factor H to bind other annexins and found that it also bound to the other 11 annexins (Fig. 1*D*). The regions of AnxA2 and Factor H involved in the protein–protein interaction are depicted in Figure 1*E*.

### Annexin A2 is expressed on the surface of different cell types in the kidney

**Figure 2:**
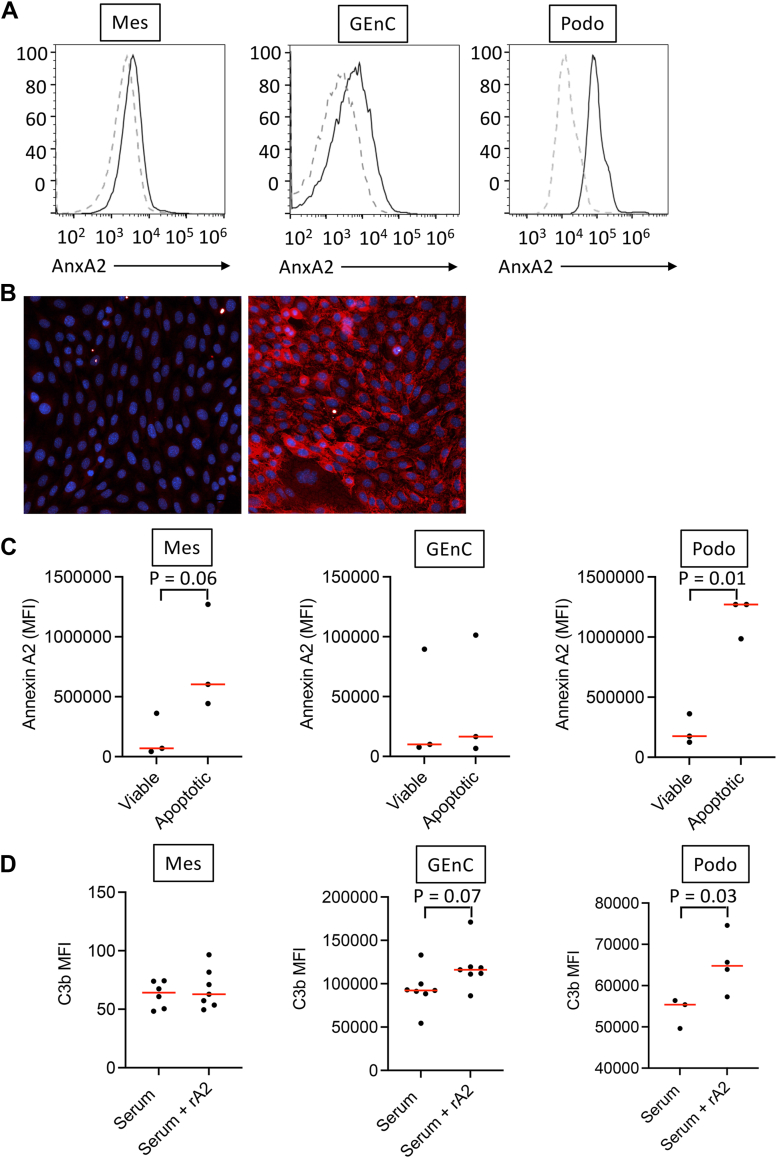
**Interactions between annexin A2 and the different glomerular cell types.***A*, expression of annexin A2 on mesangial cells, glomerular endothelial cells (GEnC), and podocytes was examined by flow cytometry. Annexin A2 was detected on all three cell types. *B*, immunofluorescence microscopy for annexin A2 in GEnCs indicated that the protein is also expressed within the cells. *C*, When injured/apoptotic and healthy cells were distinguished by side scatter and forward scatter, more annexin A2 was seen on the surface of the apoptotic mesangial cells and podocytes, whereas expression was comparable on the injured and healthy GEnC populations. *D*, when rA2 was added to glomerular cells exposed to 10% normal mouse serum, a trend towards more C3b was deposited on the surface of GEnCs compared to cells exposed to serum without rA2, and greater C3 was deposited on podocytes. Groups were compared using an unpaired Student *t* test with a two-tailed *p* value.

We examined expression of AnxA2 on various murine glomerular cell types, including mesangial cells, GEnCs, and podocytes. Using flow cytometry, AnxA2 was detected on all three cell types (Fig. 2*A*). It was also detected on the surface and within the cytosol of GEnCs by immunofluorescence microscopy (Fig. 2*B*). Gating of the healthy and injured cell populations revealed that AnxA2 was predominantly detected on injured mesangial cells and podocytes, whereas it was comparably expressed on the surface of healthy and injured populations of GEnCs (Fig. 2*C*). We previously found that the addition of rA2 to tubular epithelial cells exposed to serum increased deposition of C3 on the cells due to increased activation of the alternative pathway (22). The addition of rA2 to normal mouse serum increased C3 deposition on GEnCs and podocytes, but it did not affect complement activation on mesangial cells (Fig. 2*D*). These results may reflect differential roles for factor H, or at least for the SCR 6 to 8 region of the protein, in controlling alternative pathway activation among cell types.

**Figure 3:**
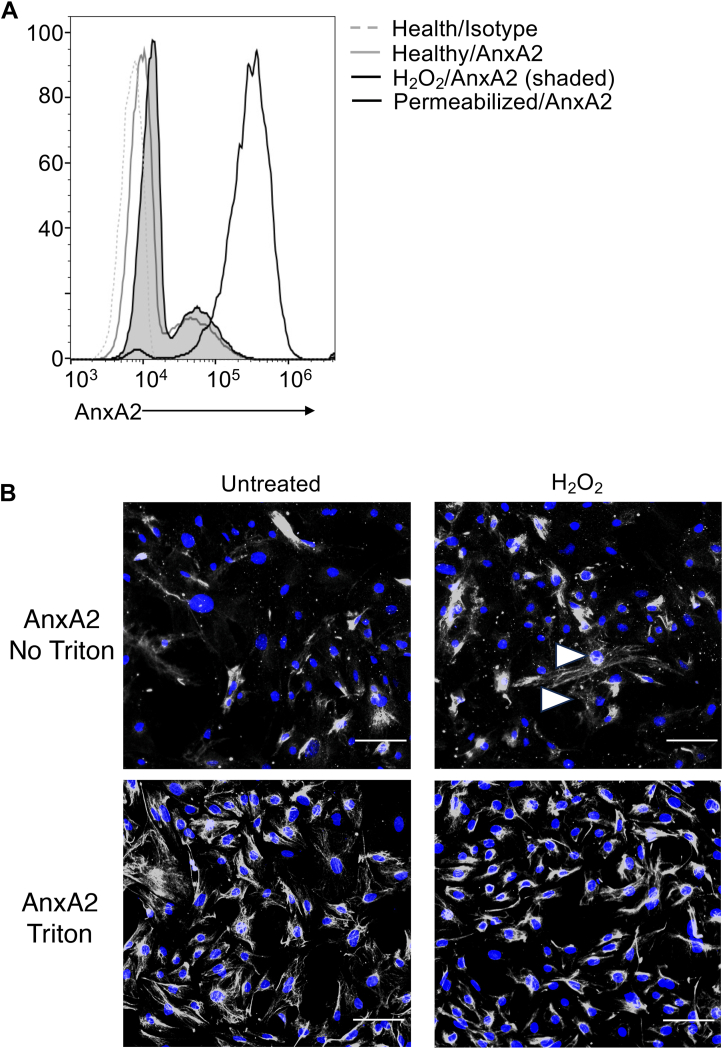
**Intracellular annexin A2 translocates to the surface of injured murine podocytes *in vitro*.***A*, annexin A2 expression on the surface of murine podocytes was examined by flow cytometry. Permeabilization of the cells increased the staining intensity, demonstrating that most of the protein is intracellular. When the cells were treated with H_2_O_2_ to cause cell injury, the expression of annexin A2 on the surface of live injured cells increased compared to live untreated cells, indicating that the protein was translocated to the cell surface. *B*, annexin A2 expression was also examined using immunofluorecence microscopy. Treatment of the cells with H_2_O_2_ increased annexin A2 staining on the surface of the cells (*arrowheads*). Permeabilization of the cells with Triton X-100 increased staining intensity, further demonstrating that most of the protein is intracellular. Cells were stained with DAPI (*blue*) and annexin A2 is shown in *gray*. Original magnification x200. Scale bar = 100 μm.

It has previously been shown that intracellular AnxA2 is sometimes translocated to the cell surface in response to injury or stress (39, 40). Permeabilization of unstressed podocytes demonstrated that most of the annexin A2 resides intracellularly at baseline (Fig. 3*A*). When the cells were injured by treatment with H_2_O_2_, we found that AnxA2 on the surface of non-permeabilized cells increased, suggesting translocation of the protein to the cell surface. Immunofluorescence microscopy of detergent-permeabilized podocytes similarly demonstrated that the majority of AnxA2 is intracellular, but that cell-surface expression of the protein increased in nonpermeabilized podocytes that were treated with H_2_O_2_ (Figs. 3*B* and S1).

**Figure 4:**
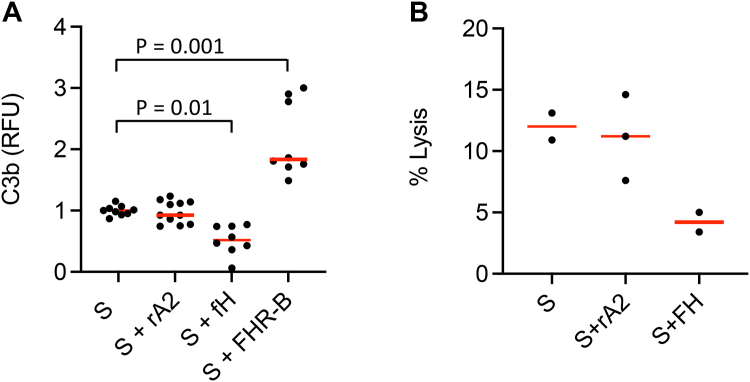
**Recombinant annexin A2 does not increase C3b deposition on Matrigel or lysis of guinea pig erythrocytes *in vitro*.***A*, enzyme-linked immunosorbent assay (ELISA) plates were coated with Matrigel, and normal mouse serum (10%) was added as a source of complement proteins. The addition of recombinant annexin A2 (rA2) did not significantly affect C3b deposition on the Matrigel-coated wells. The addition of 5 μg of recombinant Factor H reduced C3b deposition, and the addition of 10 μg recombinant Factor H related (FHR) B increased C3b deposition. *B*, guinea pig erythrocytes were incubated with normal mouse serum (10%), and the percentage of cells lysed was calculated. The addition of rA2 did not significantly increase lysis of the cells. Groups were compared using an ANOVA, with a Tukey’s multiple comparison test.

Factor H is an important regulator of alternative pathway activation on the GBM (41). We previously showed that Factor H reduces complement activation on Matrigel, as a model of the GBM, whereas the addition of murine FHR B, which we can function as an antagonist of Factor H (15), increased activation on this surface. We did not see a significant change in deposition of C3b on the Matrigel when rA2 was added to serum, indicating that rA2 does not positively regulate complement activity on this surface (Fig. 4*A*). In contrast, additional Factor H decreased activation in this assay, whereas FHR B again increased activation on the Matrigel. FHR B contains a region that is homologous to SCRs 19 to 20 in Factor H. The increased efficacy of FHR B in this assay relative to rA2 suggests that the SCR 19 to 20 region is critical for the interaction of Factor H with extracellular matrix whereas the SCR 6 to 8 region is not, consistent with a previous report (42). We also examined the effects of rA2 on the lysis of guinea pig erythrocytes by serum (Fig. 4*B*). Activation of the alternative pathway lyses these cells when they are exposed to serum because Factor H binds poorly to the cell surface. The addition of rA2 did not have a detectable effect on lysis of the cells, while additional Factor H decreased lysis. This may indicate that the SCR 19 to 20 region of factor H is more important for protecting these cells from lysis than the SCR 6 to 8 region.

### Annexin A2 promotes complement activation on glomerular endothelial cells *in vivo*

**Figure 5:**
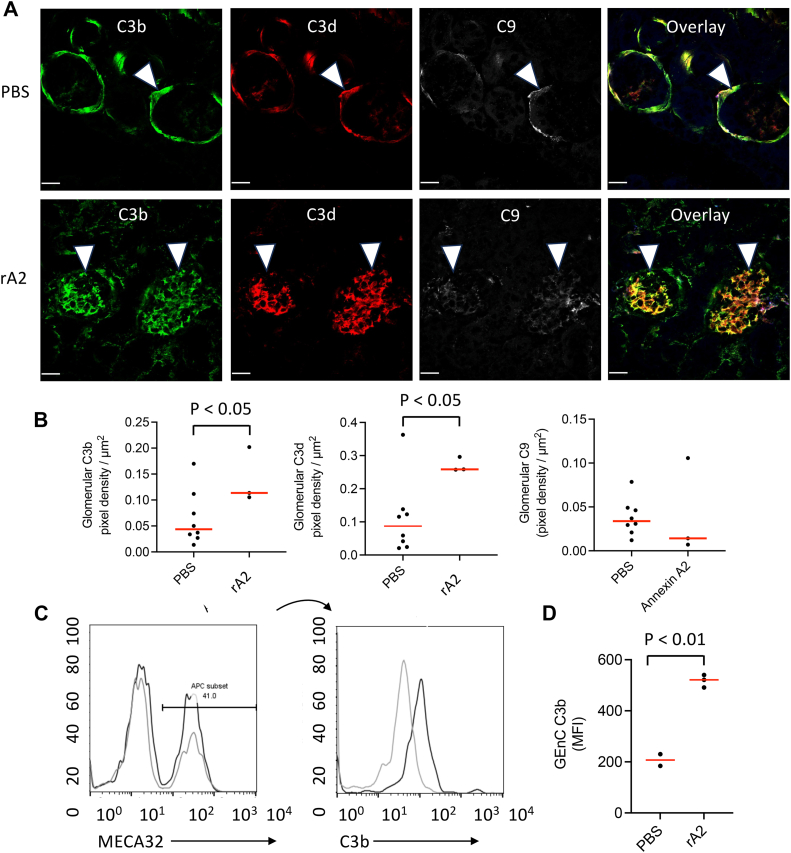
**Recombinant annexin A2 increases glomerular C3b deposition *in vivo*.***A*, *fH*^*+/−*^ mice were injected with 75 μg recombinant annexin A2. After 24 h, the kidneys were harvested and examined by immunofluorescence microscopy. Original magnification ×600. *B*, deposition of C3b and iC3b/C3d was quantified, and the abundance of both activation fragments was increased compared to mice injected with phosphate-buffered saline (PBS). Injection of the mice with rA2 did not increase C9 deposits in the glomeruli. *C*, kidneys of rA2-injected and control mice were digested with collagenase. Endothelial cells were identified by surface expression of MECA32, and C3b deposits on the cells were measured by flow cytometry. *D*, endothelial cells from rA2-injected mice had significantly more C3b deposited on their surface compared to PBS-injected mice. Groups were compared using an unpaired Student *t* test with a two-tailed *p* value.

We next tested whether AnxA2 is a positive regulator of the alternative pathway on GEnCs *in vivo.* We used mice with a heterozygous deficiency of Factor H (*fH*^*+/−*^ mice). We have previously shown that these mice are sensitive to complement dysregulation (22, 43). We injected the mice intravenously with 75 μg rA2 and harvested the kidneys after 24 h. Analysis of the kidneys by fluorescence confocal microscopy demonstrated increased deposition of C3 activation fragments in the glomerular capillaries (Fig. 5, *A* and *B*). C3 deposits in the PBS-injected mice were primarily on Bowman’s capsule and the tubular basement membrane, as has previously been described (44). We did not see increased C9 deposition, indicating that the enhanced C3 activation by rA2 does not trigger activation of the terminal pathway. To confirm that injection with rA2 caused complement activation on GEnCs *in vivo*, we generated single-cell isolates from the glomeruli of injected mice and examined the cells by flow cytometry. We gated on endothelial cells by gating the cells for MECA32, an endothelial cell marker (45), and found that MECA32+ cells had greater deposition of C3b on their surface after injection with rA2 compared to mice injected with phosphate-buffered saline (PBS) (Fig. 5, *C* and *D*).

Factor H also binds to surfaces at its carboxy-terminus *via* SCRs 19 to 20, and a recombinant murine protein that encompasses this region (rH19-20) blocks this interaction (46, 47). We injected *fH*^*+/−*^ mice with 30 μg of rH19-20 and analyzed the kidneys. Deposition of glomerular C3 fragments was similar in mice injected with rH19-20 compared to mice injected with vehicle control (Fig. S2). Comparison of the results using rA2 (which blocks SCRs 6-8) and rH19-20 suggests that the SCR6-8 region of Factor H is involved in complement regulation by Factor H in the kidney.

### Annexin A2 deficiency attenuates cyclosporine nephrotoxicity

**Figure 6:**
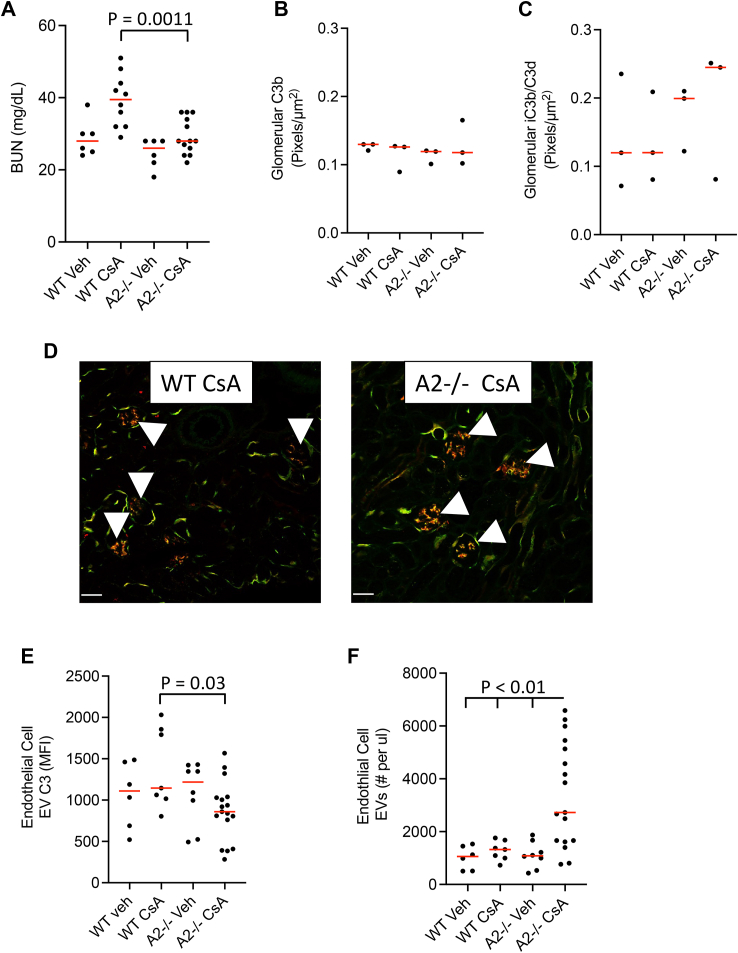
**Mice deficient in annexin A2 are protected from cyclosporine-induced kidney injury.***A*, mice with targeted deletion of the gene for annexin A2 (*A2*^*−/−*^ mice) and wild-type control mice were injected subcutaneously with cyclosporine (CsA) for 2 weeks. *A*, serum urea nitrogen (SUN) was measured as a marker of kidney function. SUN levels were significantly increased in wild-type mice injected with CsA, but did not increase in the *A2*^*−/−*^ mice. Glomerular deposition of C3b (*B*) and iC3b/C3d (*C*) were quantified by fluorescence microscopy, and the abundance of both activation fragments were similar in the wild-type and *A2*^*−/−*^ mice. *D*, representative images stained for C3b (*green*) and iC3b/C3d (*red*) are shown. Original magnification ×200. Glomeruli are indicated with *arrowheads*. *E*, endothelial extracellular vesicles (EVs) were isolated from the plasma of CsA treated mice, and C3b deposits on the surface of the EVs were measured by flow cytometry. The abundance of C3b on the EVs from wild-type mice were increased in mice treated with CsA, whereas it did not increase in *A2*^*−/−*^ mice treated with CsA. *F*, the number of endothelial EVs per μl of plasma was significantly higher in the *A2*^*−/−*^ mice treated with CsA compared to the other groups (*p* < 0.01 for *A2*^*−/−*^ CsA *versus* the other groups). Groups were compared using an ANOVA, with a Tukey’s multiple comparison test.

Cyclosporine (CsA) is a calcineurin inhibitor that is used to treat several kidney diseases, but the drug itself can also cause nephrotoxicity (48). We previously reported that CsA induces endothelial cells to release complement-activating extracellular vesicles (EVs), and that treatment of mice with high doses of the drug causes activation of the alternative pathway of complement (49). Interestingly, studies have shown that AnxA2 is bound to the surface of EVs released from the outer membranes of cells, potentially contributing to their ability to activate the alternative pathway (50). Using annexin A2-deficient mice, we examined whether AnxA2 modulates complement activation in kidneys exposed to CsA and on EVs released from the endothelial cells of treated mice. We treated wild-type and AnxA2-deficient mice daily by subcutaneous injections with the drug. After 2 weeks, blood urea nitrogen (BUN) levels were significantly lower in CsA-treated AnxA2-deficient mice compared to CsA-treated wild-type mice (Fig. 6*A*), indicating protection against loss of kidney function due to the lack of AnxA2. Glomerular C3 deposits were similar in the two strains of mice (Fig. 6, *D*–*F*).

We next analyzed endothelial EVs in the plasma of mice treated with CsA. In wild-type mice, the mean fluorescence for C3 deposition on the EVs increased after treatment with CsA, whereas C3 deposition did not increase on endothelial EVs in AnxA2-deficient mice (Fig. 6*E*). There were significantly more endothelial EVs the plasma in the AnxA2-deficient mice compared to wild-type mice treated with CsA (Fig. 6*F*). For comparison, we also examined CD41+ EVs, which predominantly come from platelets (Fig. S3). C3 deposition on CD41+ EVs did not change in either strain of mice after treatment with CsA.

Kidney ischemia/reperfusion causes alternative pathway-mediated injury of the tubules (44). We tested whether AnxA2-deficient mice were also protected in this model. We found that complement was activated in the tubulointerstitium of both wild-type and AnxA2-deficient mice after ischemia (Fig. S4*A*). Kidney injury, as assessed by BUN levels, was similar in the two strains of mice (Fig. S4*B*). Thus, although tubular epithelial cells express Anxa2, it does not appear to have a major impact on kidney injury after ischemia/reperfusion.

### AnxA2 expression in the kidney is upregulated in multiple diseases

**Figure 7:**
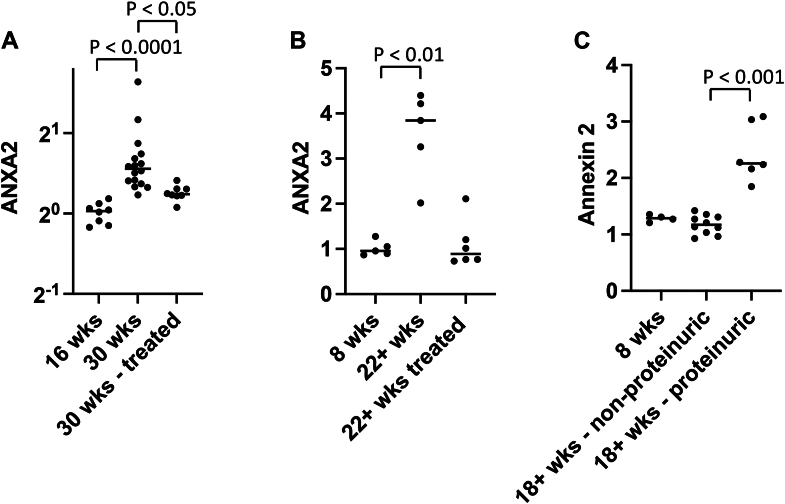
**Annexin A2 gene transcription in mice with lupus-like disease.** Kidneys from NZB/W F1, NZM2410, and NZW/BXSB mice were harvested after perfusion with sterile saline. RNA was extracted, cDNA was generated, and the transcriptional profiles of the kidneys were analyzed by microarrays. *A*, in NZB/NZW F1 female mice, annexin A2 (ANXA2) expression increased between the ages of 16 weeks (no serum autoantibodies, immune complex deposition, or proteinuria) and 30 weeks of age. Treatment of the mice with cyclophosphamide in combination with the costimulatory antagonists CTLA-4Ig and anti-CD40L induced clinical remission in the mice and was associated with decreased Annexin A2 expression. *B*, in kidneys from NZM2410 mice, expression of annexin A2 increased between 8 weeks (no autoantibodies, renal immune complex deposition, or proteinuria) and 22+ weeks of age. However, expression was not increased in mice 22+ weeks of age that were treated with a BAFF-receptor antagonist. *C*, annexin A2 expression increased in the kidneys of male (NZW × BXSB) F1 mice between 8 weeks of age (no serum autoantibodies or proteinuria) and 18+ weeks of age, but expression only increased in mice that developed proteinuria. Groups were compared using an ANOVA, with a Tukey’s multiple comparison test.

Although immune-complexes activate the classical pathway of complement, research has implicated the alternative pathway in murine models of lupus nephritis and in human disease (51, 52, 53). Using existing datasets (54, 55), we analyzed transcription of AnxA2 in three different mouse models of lupus-like kidney disease. The expression of AnxA2 was greater in proteinuric *versus* non-proteinuric NZBW mice (Fig. 7*A*), NZM2410 mice (Fig. 7*B*), and NZW/BSXB mice (Fig. 7*C*). In addition, cohorts of NZBW and NZM2410 were treated with immunomodulatory agents, and treatment of the mice was associated with decreased AnxA2 expression in the kidneys.

**Figure 8:**
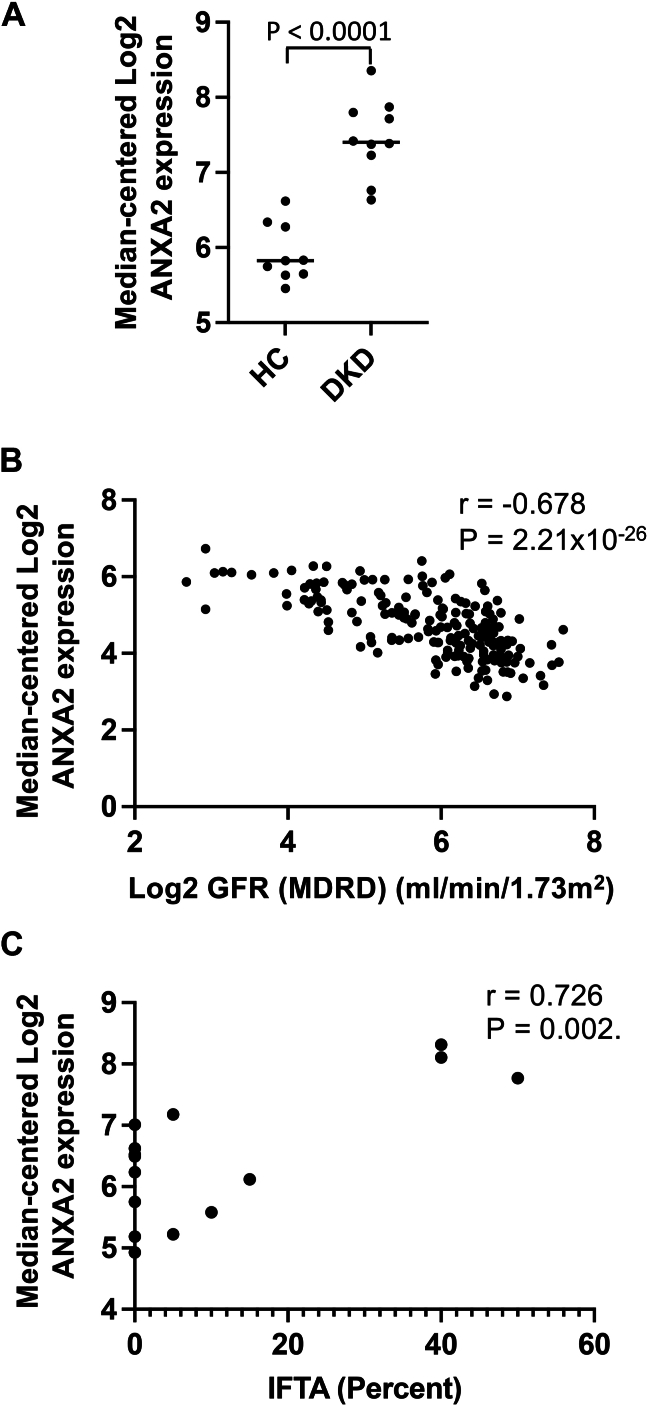
**Annexin A2 expression within the kidneys of patients with various kidney diseases.** We examined the expression of annexin A2 in the Nephroseq database, which includes analyses of biopsy samples from patients with multiple kidney diseases. *A*, analysis of *ANXA2* expression in the tubulointerstitium of patients with diabetic kidney disease demonstrated increased expression compared to healthy living donor kidneys. Groups were compared using an unpaired Student *t* test with a two-tailed *p* value. *B*, correlation of *ANXA2* expression with estimated glomerular filtration rate across the dataset reveals an inverse correlation. *C*, correlation of *ANXA2* expression with interstitial fibrosis and tubular atrophy (expressed as a percentage) in patients with focal segmental glomerulosclerosis reveals a positive correlation. Gene expression and GFR values were log2 transformed.

We also examined *ANXA2* expression in human kidney datasets available through the Nephroseq database. In patients with diabetic kidney disease (DKD), expression of *ANXA2* in the tubulointerstitium was significantly increased compared to healthy donor kidneys (Fig. 8*A*). Expression of *ANXA2* was also significantly increased in the tubulointerstitium of patients with lupus nephritis (5.401 *versus* 4.521, *p* = 0.0002) and FSGS (6.845 *versus* 5.930, *p* = 0.0502) compared to healthy controls. In several diseases *ANXA2* expression was higher in patients with nephrotic *versus* sub-nephrotic range proteinuria, including lupus nephritis (7.917 *versus* 6.049, *p* = 0.0078), IgA nephropathy (4.770 *versus* 3.885, *p* = 00079), FSGS (2.081 *versus* 1.012, *p* = 0.0146), and membranous nephropathy (2.118 *versus* 0.7312, *p* = 0.0293). The similar results seen in these various diseases suggests that increased expression of *ANXA2* is a generalized response of the kidney to injury of diverse etiologies, and *ANXA2* expression was inversely correlated with estimated glomerular filtration rate across the Nephroseq database (Fig. 8*B*). In patients with FSGS, tubulointerstitial ANXA2 expression increased in proportion to the degree of interstitial fibrosis and tubular atrophy (Fig. 8*C*).

Finally, we analyzed single nucleus RNA sequencing (snRNAseq) results and single cell ATAC sequencing (scATAC-seq) performed by the Humphrey lab on samples from patients with polycystic kidney disease and diabetic kidney disease (Fig. S5). Expression and activity of AnxA2 was higher in both diseases compared to control samples. The *ANXA2* gene was expressed by multiple different cell types in the kidneys, including epithelial cells, endothelial cells, and podocytes.

## Discussion

We previously reported that extracellular AnxA2 binds to Factor H and impairs its ability to regulate the alternative pathway (22). In the present study, we show that the third and fourth core domains of AnxA2 (part of the Ca^2+^ binding region) interact directly with SCRs 6 to 8 of Factor H. SCRs 6 to 8 of Factor H can bind to GAGs and help mediate surface recognition by the protein (16). Thus, AnxA2 may interfere with complement regulation on the surface of tissues where binding of the SCR 6 to 8 region of Factor H is required for proper complement regulation. All of the annexins contain core domains homologous to that of AnxA2, and we found that Factor H also bound to the other 11 annexins. Because alternative pathway regulation is important for protecting the glomerulus from autologous injury by uncontrolled complement activation, we next examined the effects of AnxA2 on different specific glomerular surfaces. We found that AnxA2 induces complement activation on GEnCs and podocytes *in vitro*, and it also triggers glomerular complement activation on GEnCs *in vivo* after injection into mice. Mice that lack expression of AnxA2, on the other hand, were protected from CsA-induced injury.

AnxA2 is expressed by several resident glomerular cell types (mesangial cells, GEnCs, and podocytes) as well as tubular epithelial cells. Furthermore, expression in the kidney is upregulated in multiple different diseases, including lupus nephritis, diabetic kidney disease, IgA nephropathy, FSGS, and membranous nephropathy. It is also notable that AnxA2 has been detected in glomeruli isolated from patients with C3G and in macular drusen of patients with age-related macular degeneration, disease lesions that are associated with activation of the alternative pathway (56, 57). The kidney is highly dependent on Factor H to control alternative pathway activation (58). AnxA2 expression may be a generalized response to injury or stress, and it interference with factor H may be a mechanism linking various primary insults with an inflammatory response. Intracellular AnxA2 can also be transported to the surface of cells in response to injury or cell signaling pathways (40, 59, 60), and active translocation of intracellular AnxA2 to the outer membrane may also contribute to complement activation in the extracellular milieux (summarized in Fig. S6). Because the kidney is highly dependent on Factor H to control alternative pathway activation, AnxA2 may have a stronger effect on complement regulation here than other sites throughout the body.

We previously showed that CsA induces endothelial cells to release EVs and that the CsA-associated EVs are more complement activating than constitutively released EVs (49). Other investigators have shown that subsets of EVs express AnxA2 (50). Using AnxA2-deficient mice, we show that expression of AnxA2 contributes to C3 deposition on the EV surface in mice treated with CsA. Moreover, a greater number of endothelial EVs were seen in plasma from CsA-treated AnxA2-deficient mice compared to wild-type controls. The annexins are involved in membrane repair, and the absence of AnxA2 may hinder the repair of endothelial cells after exposure to CsA, leading to release of a greater number of EVs. It is also possible, however, that C3 deposition on the EVs accelerates their clearance so that the absence of AnxA2 extends their persistence in the circulation. Regardless, kidney injury was milder in the AnxA2-deficient mice compared to wild-type controls, suggesting that AnxA2 worsens pathogenicity in this model of CsA-induced kidney injury.

AnxA2 has a wide range of reported intracellular and extracellular functions, including cell membrane dynamics, production of endosomes, packaging of miRNA into EVs, angiogenesis, and hemostasis (61, 62, 63, 64, 65). Consequently, production of AnxA2 by cells in the kidney undoubtedly elicits other responses in addition to its impact on complement regulation. AnxA2 is also the target of autoantibodies in several diseases affecting the kidneys, including anti-phospholipid antibody syndrome, lupus nephritis, and idiopathic nephrotic syndrome (34, 66, 67, 68). It is not clear whether positive regulation of the complement system by AnxA2 potentiates the inflammatory effects of these antibodies, or whether these are independent phenomena.

Our study has several limitations. We found that all 12 of the annexins bind to Factor H, and it is possible that other annexins (or combinations of them) have a stronger effect on complement activation than that of AnxA2. Along the same lines, the five FHRs contain binding regions homologous with SCRs 6 to 7 of Factor H (17). We have not examined whether AnxA2 interacts with these complement proteins, or whether such an interaction further modulates complement activation. In some diseases, such as aHUS, the SCR 19 to 20 region of factor H that is critical for controlling complement activation in the kidney (69). As AnxA2 does not affect this interaction, local expression of the protein might not have a role in complement dysregulation. It is also worth noting that the concentrations of annexin used in our *in vitro* and *in vivo* experiments were higher than is normally present in plasma, so the complement-activating effects are likely stronger than what occurs naturally (70). Glomerular diseases are generally chronic; however, so even modest imbalances in complement regulation may have an important long-term impact on disease progression (71). Further, the production of AnxA2 by cells in the kidney probably creates local concentrations of the protein that are higher than concentrations seen in serum.

In conclusion, we have found that AnxA2 is a positive regulator of the alternative pathway of complement in the kidney. The alternative pathway is activated in patients with a diverse range of glomerular diseases, many of which are associated with increased local expression of AnxA2. AnxA2 may antagonize the reno-protective effects of Factor H, and this may be an important mechanism by which cellular stress or injury engages the complement system. Given the pathogenic role that the alternative pathway plays in different kidney diseases, therapies that reverse these effects of AnxA2 may protect the kidney from complement-mediated injury. Furthermore, such a strategy might reduce complement activation in the kidney while leaving the protective functions of complement intact systemically.

## Experimental procedures

### Antibodies and other reagents

Antibodies used in the experiments included a rabbit polyclonal antibody to human annexin A2 (Proteintech; #11256-1-AP, 1:250 dilution), IgG1 monoclonal anti-AnxA2 clone Z014 (Invitrogen; #034400, 2 μg per sample), IgG1 isotype control clone MG1-45 (Abcam; # ab18448, 2 μg per sample), a Cy3-conjugated polyclonal goat anti-mouse IgG (Jackson Immuno; 1:100 dilution), an Alexa Fluor Plus 647 labeled goat anti-mouse IgG (Invitrogen; #A32728, 1:1000 dilution), a FITC-labeled goat polyclonal antibody to mouse C3 (MP Biomedicals; #55500; 10 μg/ml for tissue staining), Digoxin (DIG) labeled anti-mouse C9 (generously provided by Cees van Kooten, Leiden University Medical Center; 2 μg/ml for tissue staining), DyLight 405 IgG fraction monoclonal mouse anti-Digoxin (Jackson Immuno; #200-472-156, 2.7 μg/ml for tissue staining), and MECA32, a pan-endothelial cell antigen antibody (Novus Biologicals; NB100-77668, 0.25 μg/100 μl reaction containing 10^6^ cells). A monoclonal mouse anti-body to iC3b/C3d was produced as previously described (72). It was conjugated with AlexaFlour647 (ThermoFisher) and used at a concentration of 10 μg/ml for tissue staining. Purified annexins were purchased from CD BioSciences.

### Production of recombinant proteins

Recombinant AnxA2 and Factor H were produced as previously described (22). Briefly, cDNA for AnxA2 was reverse transcribed from the total mRNA of a C57BL/6 mouse kidney. The cDNA was inserted into the pSecTag2 Hygro B vector (Invitrogen). Recombinant murine Factor H was generated from the codon-optimized (*Homo sapiens*) DNA sequence for murine Factor H (comprising residues 19–1234; Uniprot identifier: P06909) that was subcloned into the pDONR221 entry vector (Life Technologies; construct purchased from GeneArt) along with IgG leader sequence and a histidine tag at the amino terminus. The synthetic gene was then re-combined into a pcDNA3.2/V5-Dest expression vector using Gateway LR clonase II enzyme mix (Life Technologies Inc) according to the manufacturer’s instructions. Plasmids were transfected into Chinese hamster ovary (CHO) cells and proteins were purified from the supernatant using a HiTrap His column (Cytiva).

To produce fragments of annexin A2, gene inserts of the desired regions were synthesized by Life Technologies then placed with his tag into a pCDNA3.2 vector for mammalian expression. Fragment 1–2 included the first 181 amino acids, fragment 2–3 included amino acids 104–264, and fragment 3–4 included amino acids 241–399. The plasmids were transfected into Freestyle HEK293 cells for transient expression. The proteins were purified using a Cytiva Excel column Global Life Sciences Solutions. After elution, the proteins were concentrated in MiliporeSigma Amicon tubes (MiliporeSigma Amicon) then buffer exchanged into DPBS pH 7.4. FHR B and rH19-20 were produced as previously described (15, 46). The other Factor H fragments were produced as previously described (73). Briefly, DNA sequences encoding the appropriate segments of the protein were cloned into the *Pichia pastoris* expression vector pPICZα (Invitrogen).

### ELISA-based binding assays

To examine solid phase binding of AnxA2 and Factor H, 800 ng of rA2 was adsorbed overnight to round bottom plates (Immulon). The plates were then blocked with 3% milk in tris-buffered saline (TBS), ph 7.5 for 2 h. Factor H (100 μl at 20 μg/ml in TBS with 2 mM Ca) was added to the wells and the plates were incubated for 3 h on a rocker at room temperature, and the plates were washed three times with TBS. Bound Factor H was then detected by incubating the plates with 100 μl/well of goat anti-Factor H (Quidel) diluted 1:10 in 3% milk blocking buffer for 1 h with rocking. The plates were washed five times, and 100 μl per well 1:1000 anti goat-HRP in 3% milk was added to each well. After incubating the plates for 1 h the plates were again washed and were then developed with 1-Step Ultra TMB-ELISA Substrate (Pierce). After ∼20 min the reaction was stopped with 100 μl 0.2 M sulfuric acid and absorbance was read at 450 nm.

### Guinea pig erythrocyte lysis assay

This assay was adapted from the hemolytic protection assay described by Schmidt *et al.* (74). Erythrocytes were isolated by adding 0.5 ml of whole guinea pig blood in Alsevers solution (Colorado Serum Company) to 12 ml cold wash buffer *A*, containing 20 mM HEPES (MilliporeSigma), 145 mM sodium chloride, 0.1% (w/v) gelatin from porcine skin (MilliporeSigma), and 10 mM ethylenediaminetetraacetic acid (EDTA) at pH 7.3, gently inverted and centrifuged for 10 min at 500*g*, 4 °C. The supernatant and buffy layer were discarded. This process was repeated once, followed by 2 more washes in cold wash buffer *B* (20 mM HEPES, 145 mM sodium chloride, 0.1% [w/v] gelatin, pH 7.3). Isolated GP_Er_ were then resuspended in 1 ml cold wash buffer *B*.

Hemolytic reactions were prepared on wet ice by the addition of 30% C57Bl/6 mouse serum, assay buffer (20 mM HEPES, 141 mM sodium chloride, 0.1% gelatin [w/v], and 5 mM Mg-EGTA at pH 7.3), 5 mM Mg-EGTA, sterile phosphate-buffered saline (PBS), and experimental GP_Er_ suspension at a ratio of 15:1:1:4:4. Equal volumes of the resulting mixture and various dilutions of either FH, FHR B, FHR B, rH19-20 (0.125–25 μM), or PBS were combined to a total volume of 25 μl/reaction. Heat-inactivated serum was used as a negative control. All reactions were performed in duplicate. Samples were incubated in a 37 °C water bath for 45 min, with brief agitation every 15 min. Hemolysis was stopped by the addition of 75 μl cold quenching buffer (20 mM HEPES, 145 mM sodium chloride, 5 mM EDTA, pH 7.3), followed by centrifugation at 1500*g* for 5 min at 4 °C. Supernatants were transferred to a 96-well plate and read in triplicate at *A*_*415*_, for which the average value was used to calculate the percent hemolysis. Data are presented as hemolysis relative to 100% lysis by water and are comprised of a minimum of four experimental replicates.

### *In vitro* complement activation assays

Several different cell lines were used in experiments. Immortalized murine mesangial cells (MES-13 cells) were obtained from ATCC and grown as previously described (43). Immortalized murine glomerular endothelial cells were kindly provided by Dr Michael Madaio (75). The endothelial phenotype was confirmed by flow cytometry after staining the cells for CD31, VE-cadherin, and MECA32 (data not shown). Murine podocytes were isolated from male C57BL6 mice and immortalized as previously described (76). The phenotype of the cells was confirmed by immunofluorescence after staining the cells for podocin and Wilms tumor 1 (WT1) (76). All cell lines were tested for *mycoplasma*. Cells were detached from tissue culture plates using Accutase (Innovative Cell Technologies,).

To examine the effects of the AnxA2 on complement activation, the cells were incubated with rA2 as above, and then they were exposed to 10% normal mouse serum at 37 °C for 40 min. The cells were then washed, and C3 deposition was evaluated by staining the cells with a FITC-labeled goat anti-mouse C3 antibody (MP biomedicals) diluted 1:250 in PBS with 1% fetal calf serum, followed by a donkey anti mouse IgG (Jackson Immuno Research). Fluorescence was measured on an Auora flow cytometer (Cytek Biosciences). The results were analyzed using FloJo v10.6 and v10.10 software (BD Biosciences).

To examine translocation of AnxA2 to the surface of podocytes after injury, the cells were grown to ∼85 percent confluency in T175 flasks for at least 14 days. Hydrogen peroxide was added to the media at a final concentration of 50 μM, and and cells were then incubated for 2.5 h. Cells were then washed with DPBS and detached using 2.5 ml of 0.5% Trypsin with EDTA. The cells were washed with PBS and stained with LIVE/DEAD Fixable Blue Dead Cell Stain (Fisher Scientific; #50-112-1524) according to the manufacturer's instructions. Next, cells were washed and fixed using either 4% formaldehyde or a Fixation&Permeabilization Kit (BD Biosciences). After two washes, the cells were stained and analyzed by flow cytometry as described above.

Annexin expression was also examined by immunofluorescence microcopy. Cells were grown on μ-Slide 15 Well 3D slides (IbITreat GmbH; # 85105) pre-coated with collagen (Enzo Life Sciences; ALX-522-440-0050). Cells were treated with 100 μM H_2_O_2_ or with vehicle control for 2.5 h at 37 °C. Cells were then fixed with 2% paraformaldehyde for 20 min at 4 °C and blocked for 1 h at room temperature with 5% heat inactivated fetal bovine serum with 2% BSA in DPBS. For some cells, the blocking solution also contained 0.1% Triton X-100 as a permeabilization agent. Cells were incubated with primary antibody overnight at 4 °C. They were then washed and incubated for 1 h with secondary antibodies. The cells were washed, then stained with DAPI (0.1 μg/ml, Invitrogen, #D3571) for 5 min. The cells were then imaged using Nikon Ti2E inverted microscope, and images were analyzed with NIS-Elements AR (v5.42.03) and FIJI-ImageJ software platform (v21.0.7).

### Matrigel assays

Immulon 4HBX 96-well plates (ThermoFisher) were coated with Matrigel Matrix (Corning) diluted in PBS with 0.5 mM MgCl_2_ (100 μl/well). The wells were then blocked with 4% BSA. Then, 10% C57BL6 mouse serum in PBS + 1 mM Mg was added to the wells and incubated at 37 °C for 40 min, with rocking every 10 min. In some reactions FH (40 μg/ml) or rA2 (10 μg) were added to the serum. Each reaction was performed in triplicate. C3 deposition was detected using an HRP-conjugated goat anti-mouse C3 antibody (MP biomedicals) diluted 1:3000), and plates were developed as above.

### Fluorescent and confocal microscopy

Mouse kidney sections (6 μm) were dried and fixed in cold acetone for 5 min. Sections were washed twice in cold PBS and blocked with 2% IgG-free bovine serum albumin (BSA) and 5% heat-inactivated fetal bovine serum (HI FBS) in PBS for 2 h at room temperature. Detection antibodies were diluted in 1% IgG-free BSA/2% HI FBS in PBS. Tissues were incubated with antibodies overnight at 4 °C or for 1 h at room temperature followed by at least 4 washes in cold PBS. Slides were mounted and sealed with ProLong Glass Mountant (Invitrogen) and imaged in a blinded fashion with an Olympus FV1000 inverted confocal microscope (Olympus Life Science) at ×100, ×200, and ×600 original magnification. Images were converted from binary data format and quantified with Olympus FV-10ASW software (version 04.02.02.09). Quantification was performed on images collected at x100 magnification by region of interest (ROI) selection and measurement of area and fluor intensity in each specific channel. Quantified data are presented as pixel density per μm^2^.

### *In vivo* models of kidney injury

All animal procedures were approved by the University of Colorado Institutional Animal Care and Use Committee (IACUC). To examine whether AnxA2 activates complement in the kidneys *in vivo*, *fH*^*+/−*^ mice were injected retro-orbitally with approximately (75 μg) of the protein or an equal volume of PBS. Mice were sacrificed after 24 h. Kidneys from each animal were collected in Optimal Cutting Temperature (OCT) medium (Sakura Finetek) and immediately frozen in liquid nitrogen.

To induce CsA-mediated injury, 8 to 12 week-old male C57BL/6 mice (The Jackson Laboratory, Bar Harbor, ME) or mice with targeted deletion of the gene for *ANXA2* (64) were injected subcutaneously with CsA (Bedford labs, Bedford, OH; 30 mg/kg final dose) mixed in DPBS, or they were injected with an equal volume of DPBS as a vehicle control. The mice received daily injections for 14 days. At the end of the study the mice were sacrificed, and serum, plasma, and tissues were collected. EVs were isolated from plasma as previously described (49).

### Gene transcription data

Murine gene transcription data was generated from perfused kidneys from mice with three models of lupus nephritis, as previously described (54). Human gene transcription data is from Nephroseq (www.nephroseq.org) and single cell RNA sequencing data was analyzed using software generated by the Humphreys lab as previously described [https://humphreyslab.com/SingleCell/search.php, (77, 78, 79)].

### Statistical analysis

Data were analyzed and graphs were created using GraphPad Prism software (GraphPad Prism). Comparisons between two groups were performed using unpaired *t* test. Multiple group comparisons were performed by analysis of variance (ANOVA) with *post hoc* Tukey’s *t* test. Experiments with a factorial design were analyzed by ANOVA, and interaction effects and simple effects were tested. A *p*-value of less than 0.05 was considered statistically significant.

## Data availability

All data are contained within the manuscript.

## Supporting information

This article contains supporting information.

## Conflict of interest

The authors declare the following financial interests/personal relationships which may be considered as potential competing interests:

JMT is a consultant for Q32 Bio, Inc., a company developing complement inhibitors. He also holds stock in the company. C.Q.S., is an inventor of patent applications that describes the use of complement inhibitors for therapeutic applications. C.Q.S. has received research funding from pharmaceutical companies. C.Q.S., received honoraria for speaking at symposia organized by Alexion Pharmaceuticals, Sobi and Sanofi. C.Q.S. served on an advisory committee to Sobi.
