# Supplementary material for: Annexin A2 interferes with complement regulation within the glomerulus
Source: J Biol Chem. 2025 Aug 30;301(10):110657. doi: 10.1016/j.jbc.2025.110657 (PMC12493207; doi:10.1016/j.jbc.2025.110657)
Supplement: Supporting Figures [file mmc1.pdf]

Supplemental Figure 1

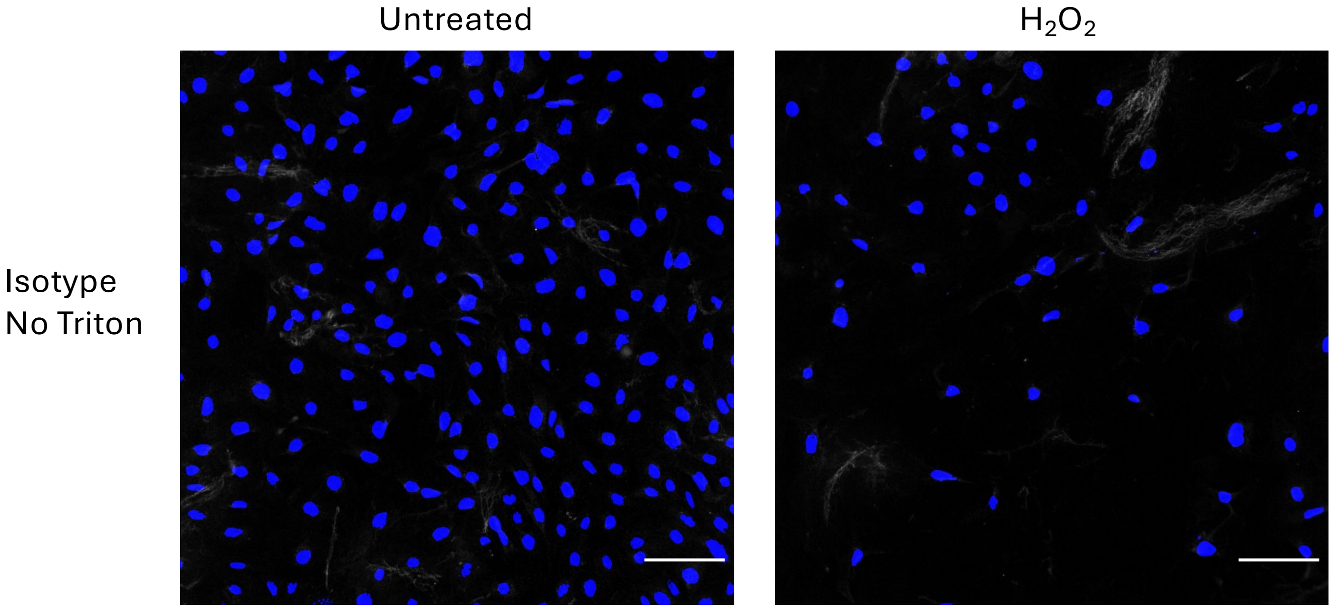

Supplemental Figure 1. Isotype control staining of murine podocytes. Murine podocytes were stained with DAPI (blue) and an isotype control antibody (shown in gray). Cells were either untreated, or they were treated with H_2O_2.
Original magnification x200. Scale bar = 100 μm.

Supplemental Figure 2

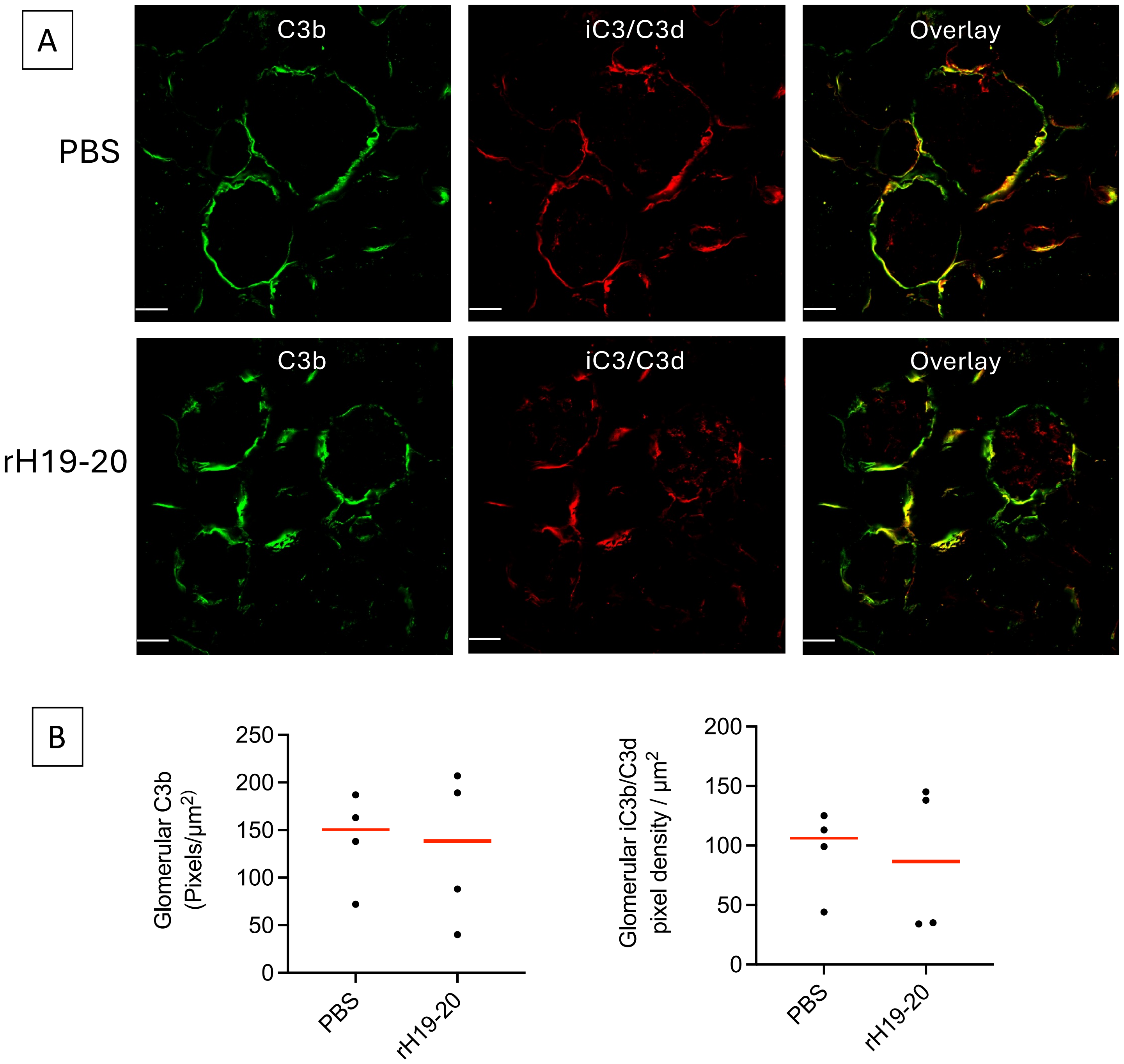

Supplemental Figure 2. Recombinant short consensus repeats 19-20 of Factor H (rH19-20) does not increase glomerular C3b deposition in vivo. A) *fH*$^{+/-}$ mice were injected with 30 μg rH19-20. After 24 hours, the kidneys were harvested and examined by immunofluorescence microscopy. C3b is shown in green, and iC3b/C3d is shown in red. Original magnification x600. Scale bar = 20 μm B) Deposition of C3b and iC3b/C3d were quantified, and the abundance of both activation fragments were similar in the rH19-20 and phosphate buffered saline (PBS) injected mice.

Supplemental Figure 3

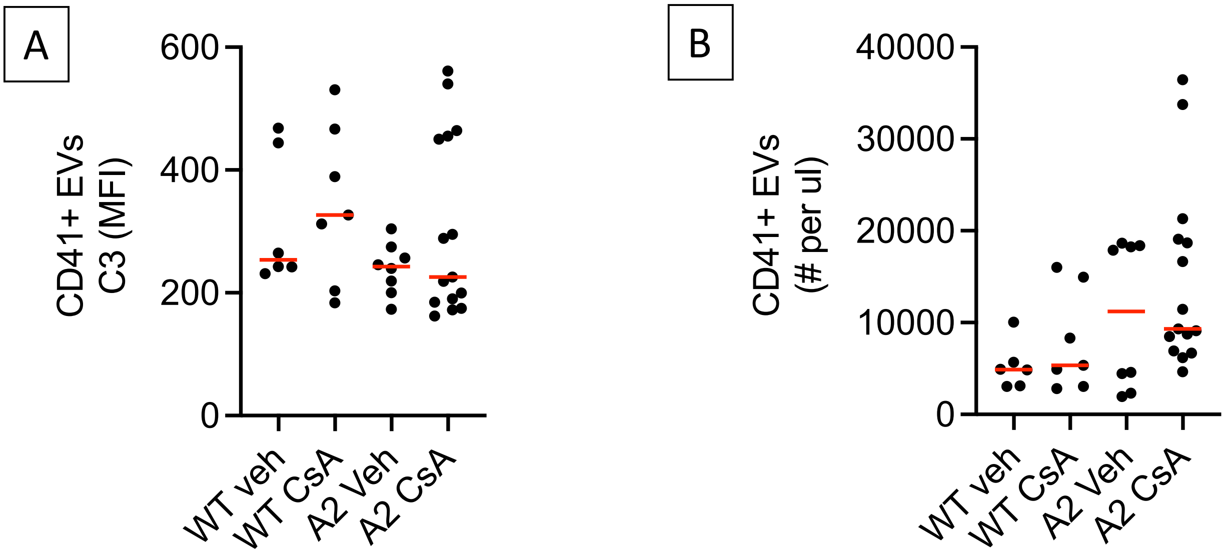

Supplemental Figure 3. CD41+ Extracellular vesicles (EVs) in the plasma of wild-type and annexin A2-deficient mice injected with cyclosporine (CsA). A) Mice with targeted deletion of the gene for annexin A2 (*A2*$^{-/-}$ mice) and wild-type control mice were injected subcutaneously with cyclosporine (CsA) for two weeks. A) CD41+ EVs were isolated from the plasma of the mice, and C3b deposits on the surface of the EVs were measured by flow cytometry. The abundance of deposited C3b was similar in all of the groups. B) There was a trend towards a greater number of CD41+ EVs per µl of plasma in samples from the *A2*$^{-/-}$ mice, but the differences between groups was not statistically significant.

Supplemental Figure 4

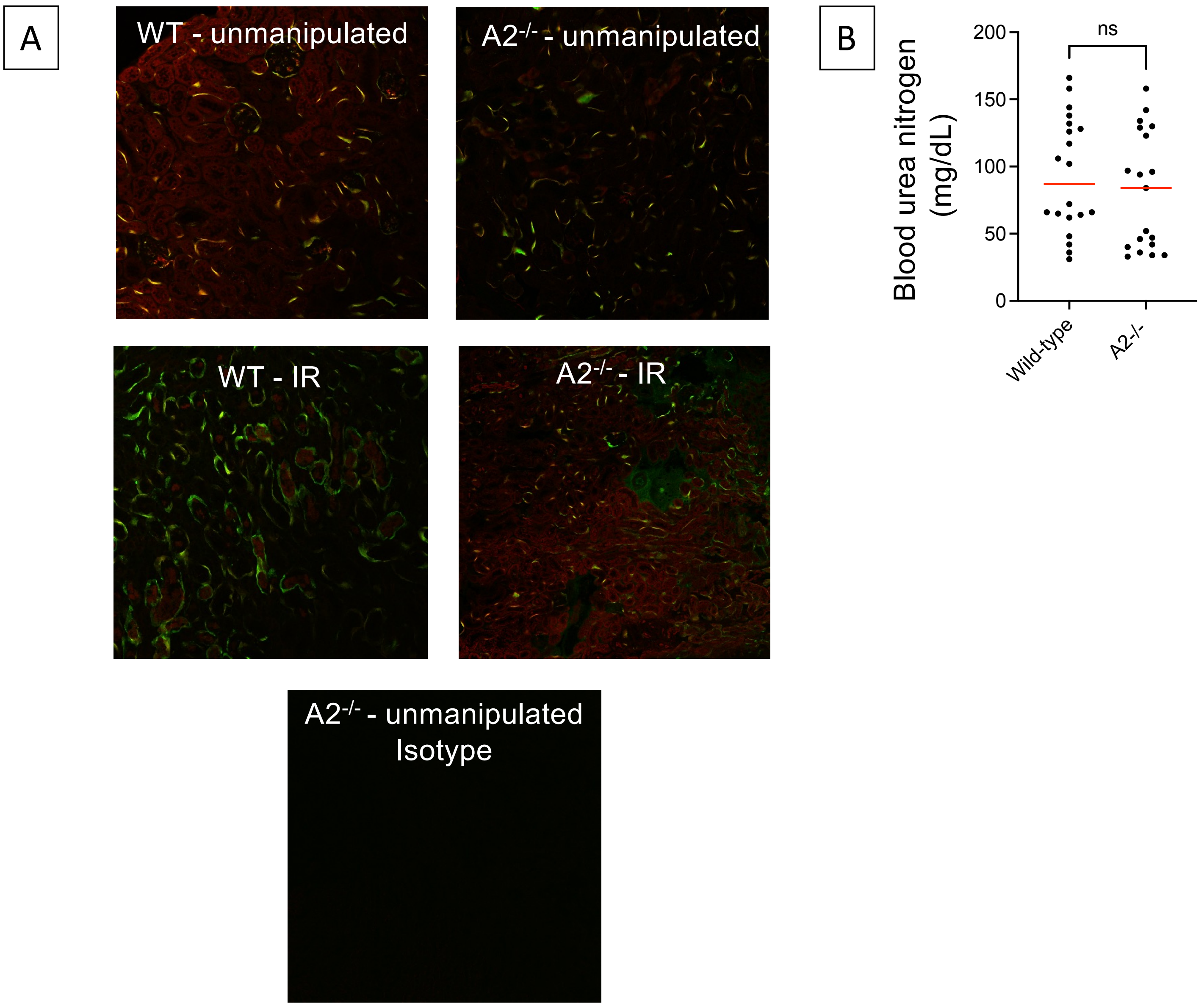

Supplemental Figure 4. Kidney ischemia/reperfusion of C57BL/6 and annexin A2$^{-/-}$ mice. C57BL/6 wild-type and mice with targeted deletion of the gene for annexin A2 (*A2*$^{-/-}$ mice) were subjected to 24 minutes of bilateral kidney ischemia and 24 hours of reperfusion. A) Immunofluorescence miscoscopy of the kidneys for C3b (green) and iC3b/C3d (red) deposition shows scattered tubulointerstitial deposits in the kidneys of both strains of mice. C3 fragment deposition was more extensive in both strains after ischemia/reperfusion. B) Serum urea nitrogen (SUN) levels were elevated after ischemia/reperfusion. Comparison of the two strains of mice did not show a significant difference.

Supplemental Figure 5

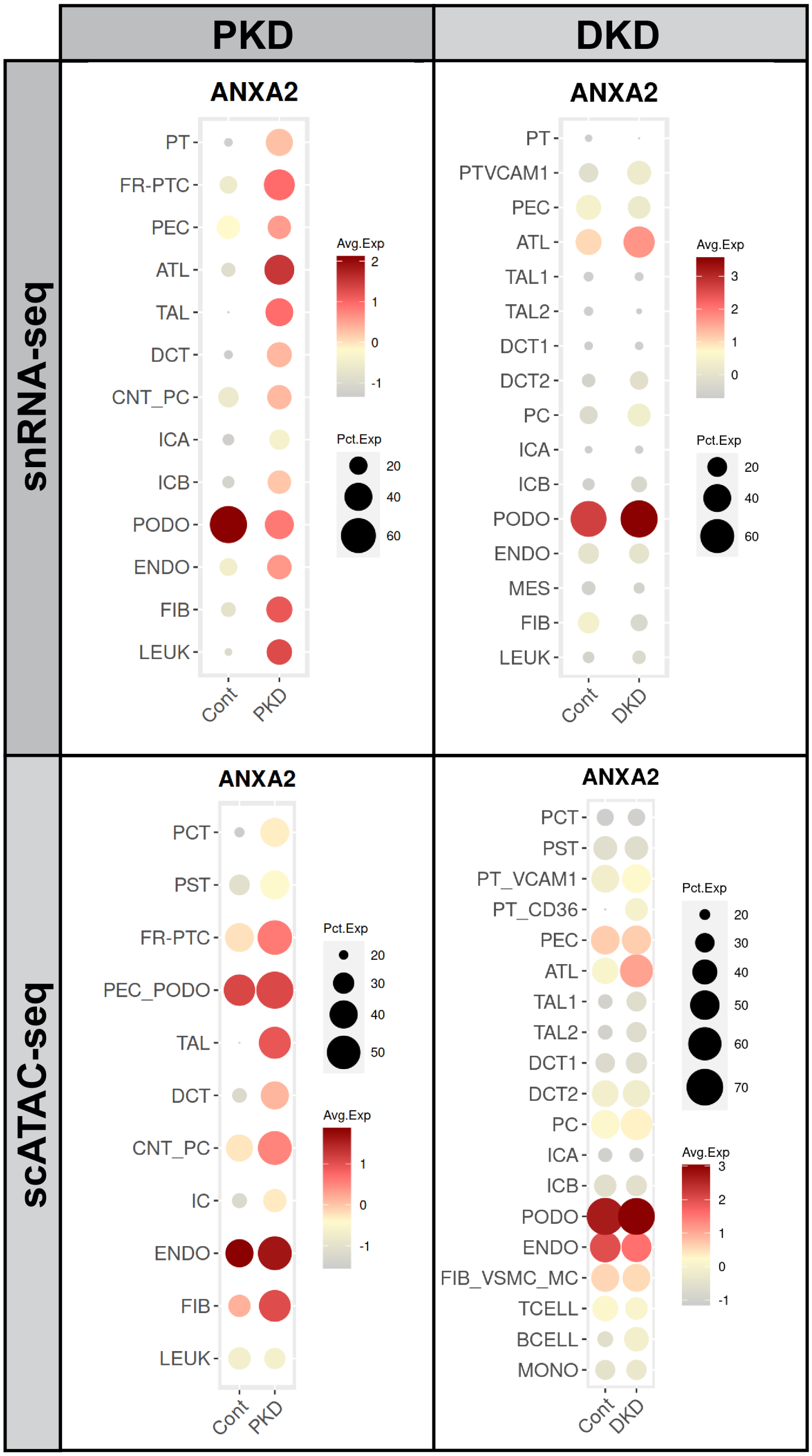

Supplemental Figure 5. Single nuclear RNA sequencing (snRNAseq) and single cell ATAC sequencing (scATAC-seq) data for annexin A2 expression and activity in kidneys from patients with polycystic kidney disease (PKD) and diabetic kidney disease (DKD). snRNAseq and scATAC-seq data from the Humphreys lab was examined to determine the patterns of annexin A2 expression in PKD and DKD kidneys. Expression was increased in kidneys with both diseases. Expression was seen in multiple different kidney cell types, including glomerular endothelial cells, podocytes, and tubular epithelial cells.

Supplemental Figure 6

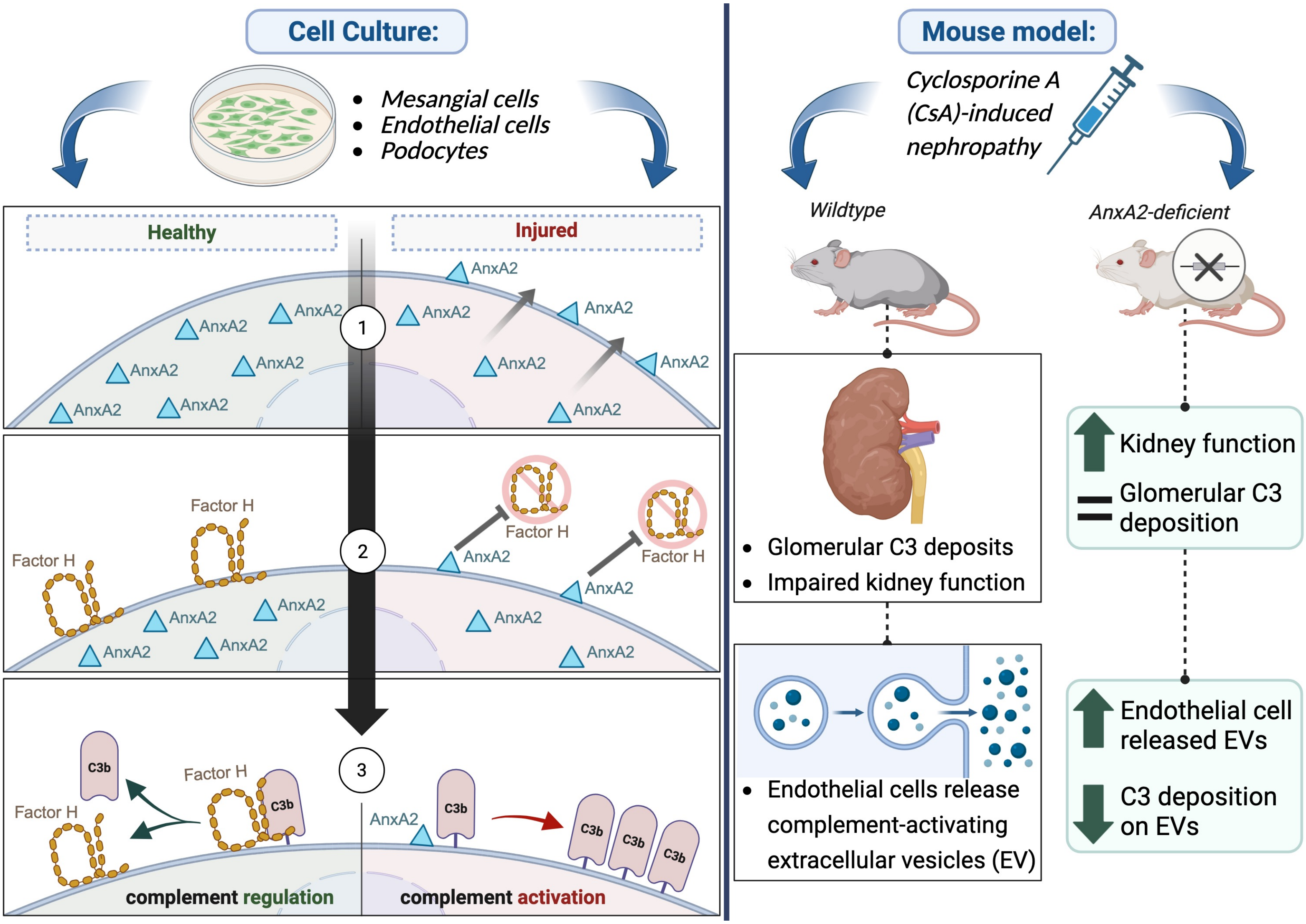

Supplemental Figure 6. Summary of findings. In vitro experiments showed that injury of cells causes translocation of annexin A2 to the cell surface. Annexin A2 blocks complement regulation by factor H, thereby promoting C3b deposition on the cell surface. In vivo experiments showed that treatment of mice with cyclosporin (CsA) causes endothelial cells to release extracellular vesicles (EVs). In annexin A2-deficient mice, complement activation on the EVs is reduced, and the mice are protected from kidney injury.
